# SHARING INSIGHTS: INTRODUCING BRAIN HEALTH RESEARCH TO LA COMUNIDAD LATINA IN NORTH CAROLINA

**DOI:** 10.1093/geroni/igae098.1407

**Published:** 2024-12-31

**Authors:** Marianne Chanti-Ketterl, Kathleen Welsh-Bohmer, Sofia Royo, Brenda Plassman

**Affiliations:** Duke University, Durham, North Carolina, United States; Duke University, Durham, North Carolina, United States; Duke University, Durham, North Carolina, United States; Duke University Medical Center, Durham, North Carolina, United States

## Abstract

Alzheimer’s disease disproportionally impacts the Hispanic/Latino(a)x (hereafter Latino) community. Ensuring Latino representation in brain health research is essential for understanding how brain disorders affect this diverse population. However, recruiting Latinos into research studies presents multifaceted challenges, including structural inequities, cultural beliefs, values (i.e. faith and family beliefs), and immigration status. Within the North Carolina Registry for Brain Health (NC Registry), we have achieved a twofold increase in Latino enrollment within a year by effectively utilizing existing resources and targeting upstream factors, such as diversifying staffing and collaborators. Increasing the diversity of the NC Registry offers research opportunities to a broader group and supports researchers’ efforts to enroll a diverse sample. During this symposium, we will present strategies employed to effectively involve Latinos in the NC Registry, educational initiatives focusing on brain health, and various forms of community engagement. Additionally, we will delineate the pathway from NC Registry participation to enrollment in research studies and elucidate the participant tracking methodology employed. This methodology is key to ensuring that individuals are contacted for the appropriate studies and are not overburdened with multiple requests in a short period of time. Furthermore, we will discuss how this foundational work contributes to fostering community trust and increasing interest in engaging in research studies supported by the Duke-UNC Alzheimer’s Disease Research Center. We will conclude by offering recommendations for recruiting and retaining Latinos in brain health research.

